# The New Zealand System Level Measures Programme – a New Policy to Implement a Whole of System Performance Framework Using Health Alliances

**DOI:** 10.5334/ijic.9043

**Published:** 2025-11-26

**Authors:** Kanchan M. Sharma, Peter B. Jones

**Affiliations:** 1 Te Tai Ōhanga –The Treasury, 1 The Terrace, Wellington 6011, NZ; 2 Department of Medicine, Faculty of Medical and Health Sciences, University of Auckland, 34 Princes Street, Auckland CBD, Auckland 1010, NZ

**Keywords:** System Level Measures Programme, integrated care, collaborative networks, alliances, health system leadership, system performance

## Abstract

**Introduction:**

In 2016, the New Zealand Ministry of Health (MoH) introduced a whole of system performance policy known as the System Level Measures (SLM) programme to deliver integrated care using health alliances. Alliances were trust-based collaborative networks introduced in 2013 to integrate the planning and delivery of health care between primary care and hospital settings. The SLM programme attempted to move away from narrow target-based and pay-for-performance approaches focused on single organisations to a shared responsibility and decision-making approach using alliances.

**Description:**

The SLM programme was co-designed by the MoH and health sector clinicians, analysts, and managers. It consisted of six system level measures, each supported by a suite of contributory measures. System level measures were outcome focused while contributory measures focused more on process and activity. Alliances were responsible for leading the implementation of the SLM programme in their districts. Implementation of the programme required alliances to share health information and resources, identify priorities for their district, agree an improvement plan, and commit to delivering it. The MoH assisted the implementation process, provided access to data, approved the plan, monitored progress against the plan, and administered incentive funding for Primary Health Organisations. At the end of each year, alliances were expected to review and reflect on their successes and failures to inform the following year’s plan.

**Discussion:**

Success with implementation of the programme varied and was influenced by two key factors. First, there was a lack of sponsorship from the centre. This meant that although there was sector support for the programme, there was a lack of leadership and adequate resourcing from the centre to sustain the programme. Second, the MoH expected alliances to use the SLM programme to improve their local relationships, develop their capacity and capability for improvement and improve their maturity as a network. Reflection and evaluation of the SLM programme found that these were necessary pre-conditions for alliances to succeed with implementation of the programme. In the end, this improvement programme could not be reconciled with an accountability framework.

**Conclusion:**

New Zealand’s SLM programme was a unique experiment with a new system performance framework to improve integration across the health system. Its implementation provides important lessons on the role of centre to create the right conditions for integrated care initiatives, such as the SLM programme, to succeed. We conclude that successful implementation of integrated care initiatives requires sponsorship and leadership from senior leaders, adequate and appropriate resourcing, right incentives, and most importantly a strong platform for a collaborative way of working which nurtures high-trust relationships among system leaders and continuous learning.

## Background

This paper describes a system performance framework, known as the System Level Measures (SLM) Programme, introduced in the New Zealand (NZ) health system to enhance system integration and shift away from a narrow target-based accountability approach. The aim of the SLM programme was to use a whole of system approach to improve performance and health outcomes using clinically led continuous quality improvement. The SLM programme was implemented using collaborative networks, known as alliances, that existed in the NZ health system. This policy was a first attempt to develop and implement a national whole of system performance framework using a shared responsibility model. Key elements of the SLM programme included a shift away from output to health outcome measures, delivery of integrated patient care using a bottom-up improvement approach, incentivising quality improvement and collaborative behaviours between primary and hospital care, sharing and using patient data to develop improvement actions, and a continuous learning approach.

Much about the development of the SLM programme is not published, and the guidance documents, plans, measure details and data relating to the programme are no longer available on the NZ Ministry of Health (MoH) website. The information presented in this paper uses knowledge of the authors (KMS and PBJ) who were involved in the development of the SLM programme. The paper reports on the development of the SLM programme, its success, and lessons for policymakers.

At the time when this policy was introduced, the NZ health system had 20 geographically based District Health Boards (DHBs) that delivered publicly funded hospital and specialist services and purchased primary care services from Primary Health Organisations (PHOs). DHBs funded PHOs (not-for-profit meso-layer organisations) to provide comprehensive primary care services through their member general practices. Citizens chose the general practice to enrol with and general practices chose which PHO to become a member of [1]. The MoH had overall leadership of the health and disability system. A simplified visual description of the NZ health system at the time of this research is shown in Supplementary Figure A.

The New Public Management (NPM) doctrines [2], introduced in the NZ public sector organisations in the 1980s and 1990s, led to the introduction of target-based national accountability measures for DHBs and PHOs [3,4,5].

For PHOs, a pay-for-performance programme, known as the PHO Performance Programme (PPP), began in 2005. PPP aimed to improve the health of enrolled populations, reduce health inequities and reward quality improvement within PHOs [4]. It was supported by weighted incentive payments (from a total pool of approximately NZ$23 million) paid to PHOs quarterly for achieving about 30 targets. The payments were weighted for higher need populations (Māori and Pacific populations, and those living in the most socioeconomically deprived areas) [4].

From 2009 to 2017, six national health targets were used to publicly report DHB performance, published in major newspapers and on the MoH website in a league table. The six targets were: improved access to elective surgery, shorter stays in emergency departments, shorter cancer treatment times, better help for smokers to quit, increased immunisation for babies aged eight months, and raising healthy kids (at B4 School Check, caregivers of four-year-olds with body mass index above the 98^th^ centile offered referral to family-based nutrition, activity, and lifestyle intervention). PHO performance was also publicly reported for two of the health targets: increased immunisation for babies by age eight months, and better help for smokers to quit. Successive governments during this period used the six targets as a measure of health system performance. The implementation of targets was supported by the MoH using target champions, data collection and reporting, performance monitoring and advice to Ministers and Cabinet. Failure to achieve the national targets resulted in financial, reputational, and other sanctions for DHBs, and PHOs and their providers, including letters of expectations from the Minister of Health to DHB chief executives.

PPP had some success, for instance in achieving high rates of childhood immunisation that were equitable for Māori but led to some unintended consequences and poor behaviours. For example, a narrow focus on achieving health targets meant other important population and personal health areas were neglected; districts aimed to be safely in the middle of the league table rather than striving for improvement; districts with populations with higher needs became disillusioned and disengaged as they could never achieve the targets and lost out on incentive funding, cementing their place in the league; districts would ‘game’ the measures or do the minimum in order to still get their incentive money; it was a management-led process, largely in secondary care, in which clinicians could easily avoid genuine engagement. The General Practice Leaders Forum (GPLF) wanted to move away from the accountability approach of PPP and develop a meaningful system performance programme for primary care with measures that incentivised integration of primary and hospital care.

### Alliances

Since 2013, the MoH contractually required DHBs and PHOs in each district to form alliances. The alliancing model, adapted from the construction industry, was introduced in the NZ health system by Canterbury DHB [6]. The model was underpinned by the principle that multiple organisations can achieve better things by working together on pre-agreed gains and losses (‘everyone wins or everyone loses’) [6]. It is a collective contract where the focus is on the overall performance of all parties and encourages partners to help each other to achieve the pre-agreed task or work programme [6]. The alliancing model provided a platform to involve clinical leaders not holding formal leadership roles in their employer organisations in decision-making about the planning, funding and delivery of health care in their districts [7].

NZ health alliances were not legal entities and could neither commission services nor hold their own budgets for spending on health care. Instead, alliances operated with support from DHBs; members defined a work programme and agreed on a shared vision and goals for their local health system with their DHBs. Alliance partners had access to a flexible funding pool held by DHBs to deliver their work programme.

Most alliances existed solely to meet the contractual requirement and didn’t understand the philosophy behind an alliancing way of working. The contractual arrangement hindered some DHBs from including other local partners in the alliance that were not part of a PHO, such as pharmacy and maternity services. The mandate narrowed the thinking of DHB and PHO system leaders and provided a perverse incentive whereby limiting alliance membership meant that other providers could not access the flexible funding pool.

## The Integrated Performance and Incentive Framework (IPIF)

In 2013, the GPLF and the MoH commissioned an expert advisory group (EAG) made up of senior clinical and sector leaders to develop a new system performance framework. In February 2014, the EAG released its report which outlined their vision for a new framework, referred to as the Integrated Performance and Incentive Framework (IPIF). The EAG envisaged that IPIF would address quality and accessibility, encourage integration of primary and hospital care, shift the focus from outputs to outcomes, and create an environment for local, clinically-led continuous quality improvement [8]. IPIF was proposed to have a tiered incentive structure for PHOs with earned autonomy at the top. The EAG wanted to use IPIF to align the DHB accountability framework with the new PHO performance programme and create a whole of system performance framework based on an improvement philosophy [8].

Phase 1 of IPIF began with a focus on primary care, with the intent over time to broaden and include other community providers and (eventually) hospital care. From 1 July 2014, the PPP ceased and was replaced with five interim targets: more heart and diabetes checks, better help for smokers to quit, increased immunisation rates for infants aged eight months, increased immunisation for infants aged two years, and cervical screening rate [8,9]. As with PPP, PHOs were paid based on their quarterly performance. The interim arrangement was put in place to signal a shift away from the PPP and to allow the MoH to work with clinical and sector leaders to develop IPIF [8,9]. The public reporting of six health targets continued for DHBs and PHOs.

Although IPIF had many workstreams, the initial focus was on measures development. The EAG recommended the use of the ‘Triple Aim’ framework [10] to organise performance measures and to incorporate a life course approach (from conception, infancy, childhood, adolescence, adulthood and elderhood) [9]. The ‘Triple Aim’ was developed by the Institute of Healthcare Improvement (IHI) as an organising framework to simultaneously improve population health, patient experience of care, and reduce per capita cost [11].

The governance of IPIF was twofold: an internal project steering group (IPSG) that brought together senior leaders from the DHB accountability and primary care teams in the MoH, and an external joint project steering group (JPSG) that was made up of senior clinical and sector leaders. The MoH chaired the JPSG, which was tasked to develop the tiered incentive structure. The IPSG and the JPSG had different ideas on what an incentive structure would look like and how PHOs could shift along the scale of earned autonomy. There was a lack of shared vision on the goals and outcomes of IPIF between IPSG and JPSG.

At the time the IPIF was being developed, the Government had established a National Health Board (NHB), a department within the MoH, to monitor performance of DHBs. The focus of the Government and the NHB was to reduce DHB financial deficits and improve performance against health targets to assure the public on system performance. The improvement philosophy of IPIF was perceived as a ‘soft’ policy tool by the executive leadership team (ELT) at the MoH. As such, the sponsorship for IPIF was absent from the centre. This absence was demonstrated through a lack of genuine participation and leadership in the IPSG meetings where MoH ELT members mostly disagreed with sector and clinical leaders’ vision for IPIF to move away from target-based performance and resisted changes to DHB accountability framework. The cessation of IPIF led to a loss of trust and confidence by primary care senior leaders in the senior MoH leaders.

## Development of the System Level Measures programme

A change within government in 2015 (a change of Minister of Health) led to a change in direction, and development of IPIF was paused. The IPSG and JPSG were disbanded. The renewed focus of the work was on stronger alignment between primary and hospital care to deliver integrated patient care using a bottom-up improvement approach.

The life course approach was retired, and instead the direction from the Minister of Health was for the MoH to co-develop a small set of health system outcome measures that focused on children and youth, patient experience, and prevention and early detection. Between July 2015 and June 2016, the authors, using an iterative consultation process with the broader sector, produced a shortlist of twelve measures from which the Minister of Health agreed on six to be implemented under a new framework called the System Level Measures programme (the SLM programme).

The six system-level-measures (SLMs) were: Ambulatory Sensitive Hospitalisation (ASH) rates for zero to four-year olds; acute hospital bed days per capita; patient experience of care combining adult hospital and primary care patient experience surveys; amenable mortality rates; babies living in smokefree homes; and youth access to and utilisation of youth appropriate health services. The last measure did not exist and was developed and implemented later.

The SLMs aligned with the ‘Triple Aim’ and were deliberately chosen so that no one single provider in the health and disability system could sincerely (without gaming) improve the measure, no matter how efficient and high performing it was. Instead, the expectation was that primary and hospital clinicians would work together using improvement science to agree actions in an improvement plan and implement them (the original intent of IPIF). This approach would encourage locally led quality improvement actions that addressed local population health needs and that linked to the national system performance framework through a small set of health outcome measures.

The SLMs were supported by a suite of process and activity measures, known as contributory measures, which were available through an online measures library. The library was created so implementers could easily see the logic of how SLMs (the big dots) were connected to contributory measures (the little dots) and to ensure that all measures used in the programme were nationally comparable. The MoH placed responsibility for leading the implementation of the SLM programme with alliances. Alliances were expected to use SLMs to determine the focus of improvement for their districts (improvement milestones), determine local actions to achieve their improvement milestones, and then choose relevant contributory measures to monitor progress against their improvement actions.

There was an agreement with the Minister of Health that the SLM programme would be implemented as a system performance framework and there would be no public reporting of the six SLM measures. The national health targets would continue as the system accountability framework with public reporting. The SLM programme focused on continuous improvement (doing the right thing) while the health targets focused on meeting Cabinet requirements and public expectations to demonstrate efficiency and accountability of the system (was it done right). Some of the health targets were contributory measures in the SLM programme (e.g. childhood immunisation rates contributory to childhood ASH rates).

Incentive funding for PHOs that was attached to the PPP was re-purposed for the SLM programme [12]. There was a significant shift away from a pay for performance method towards one that focused on incentivising quality improvement and collaborative behaviours between primary and hospital care. Twenty-five percent of the incentive funding was paid to PHOs upfront at the beginning of each financial year (FY) to develop capacity and capability for quality improvement, 50% paid upon MoH approval of the SLM plan (end of the first quarter), and the final 25% was paid on achieving the agreed milestones for three SLMs and for achieving the two national health targets for primary care: increased immunisations; and better help for smokers to quit (first quarter of the following FY) [12].

## Implementation of the SLM programme

The implementation of the SLM programme was driven by frontline healthcare staff and middle layer health system leaders in DHBs, PHOs and the MoH. In some districts implementation also included other health system partners such as pharmacy, lead maternity carers, well child providers, Māori and Pacific providers, and youth health service providers.

The SLM programme implementation guidance stated that a high-quality plan was developed through clinically led processes involving multiple stakeholders, using evidence from health and social data, and had improvement actions expected to change health outcomes. This planning process, along with the full implementation of each plan and the ability to undertake reflective learning on processes and progress, indicated successful implementation of the programme.

Alliance leadership teams were tasked with development of an annual improvement plan using a collaborative approach underpinned by robust improvement science, and monitoring and reporting progress against successive plans.

Since alliances were not legal entities, accountability for the SLM programme sat with DHBs. The SLM plan and the quarterly reporting were part of annual DHB plans and submitted to the MoH on behalf of alliances. Some of the measures were in both plans (e.g. ASH rates) and DHBs could reference their SLM plan to meet annual plan requirements. This approach integrated the PHO performance framework in the DHB accountability framework to a certain degree but created other issues, which are discussed later in the paper.

The SLM improvement plan included improvement milestones for each of the six SLMs, the frontline improvement actions, the contributory measures, and the signatures of all alliance partner Chief Executives. The signatures on the plan were a proxy to demonstrate organisational commitment to an integrated and partnership approach to the development and implementation of the improvement plan.

The plans were assessed and approved by the MoH, and quarterly reports demonstrated alliances’ progress against the plan. At the end of the financial year, alliances reported whether they implemented the plan and achieved their improvement milestones for the six system level measures. To receive the final payment (25% incentive funding), the report had to include reflection on successes and failures using the ‘Plan, Do, Study, Act’ cycle and share insights and lessons to inform the following year’s plan.

In the first year of implementation, the MoH’s aim was to receive a plan from each of the alliances regardless of the quality of the plan. This approach recognised that the programme required clinical and operational leaders from across DHBs and PHOs to work collaboratively with frontline clinicians to implement a quality improvement programme in a way that had not been done before. Alliances could choose to maintain their performance in the first year while they examined their data to understand what was driving their rates for the SLMs. The alliance also did not have to provide their improvement actions in the national plan. These were expected to be part of their local project plans.

In the second year, the MoH raised expectations and asked that alliances seek an improvement from their past performance for the SLMs and include a brief description of improvement actions in the plan submitted to the MoH.

In the third year, the MoH emphasised the need for the plans to focus on addressing health inequities. This required involvement of Māori and Pacific teams in DHBs and a stronger line of sight between the milestones, actions, and the contributory measures. The MoH also asked alliances to extend their membership beyond DHBs and PHOs and include other partners in the system such as patients, communities, and iwi (Māori tribe), ambulance, pharmacy, and maternity.

The MoH made dynamic policy changes to the programme based on feedback received from alliances and frontline staff involved in the implementation of the programme. For example, there was a requirement for alliances to set an annual improvement milestone for all six SLMs, however feedback from sector led to change for one of the measures. Owing to time delays in releasing amenable mortality rates caused by the need for Coronial processes to be completed, alliances were required to set a three-to-five-year milestone. The MoH also co-developed the SLM for youth health with the sector and young people.

### The role of Ministry of Health

The authors (KMS and PBJ) were employed by the MoH and led the development and implementation of the SLM programme. KMS has a health management and policy background and PBJ is a practising senior clinician and academic. They provided policy advice to government to set up the SLM programme, co-developed the SLMs with the sector and were responsible for overseeing its implementation. Their role in the implementation included supporting alliances to develop their annual SLM plans, approval of these plans and monitoring alliance progress against these plans. They facilitated relationships among primary, community and hospital services, between DHBs and PHOs, within the MoH, and engaged clinicians in the implementation of the programme.

The MoH also managed the PHO incentive funding, shared identifiable patient-level data with DHBs and PHOs from national collections to support improvement actions and worked on data integrity of SLMs and contributory measures. An example was working with Well Child providers and lead maternity carers to improve data collection, integrating data they collected with hospital and PHO data, and sharing with practices for their enrolled population to inform improvement actions.

At the onset of the implementation, the MoH, appreciating the dual challenge of implementing the SLM programme compounded by a short lead-in time, adopted a long-term strategy. It was important for the MoH to demonstrate philosophies of a collaborative way of working (clinicians and managers, primary, hospital and community care) and a continuous improvement approach both to the way the programme was implemented and to programme outcomes. The long-term view was that as the programme matured and the processes became embedded, there would be an incremental increase in the quality of improvement plans and progress towards achieving medium- and long-term outcomes.

### Insights from the implementation of the SLM programme

Implementation of the programme highlighted variability in the way alliances worked to develop an improvement plan and whether they were able to fully implement it in their districts. We identified several elements and contexts that caused this variability. These elements and contexts, which were found to be pre-conditions of successful integrated care efforts, were revealed in the two evaluations of the programme discussed later in the paper. The focus of this paper is to share other lessons learnt during the implementation of the SLM programme, these relate to the contextual environment at the centre.

While the SLM programme had the support of then Minister of Health, the focus remained on DHB performance against publicly reported targets. The MoH senior leaders who advised the Minister on the DHB accountability framework did not champion the SLM programme. The programme was perceived as a primary care quality improvement programme and there was a lack of adequate resourcing in the MoH to support its implementation.

Structural issues in the health system impeded successful implementation of the SLM programme. The DHB-PHO contractual arrangements impacted alliance memberships and access to the flexible funding pool to support the implementation of the SLM improvement plan. DHBs perceived alliances as a way to manage PHOs in their districts and PHOs saw alliances as a mechanism to access the flexible funding pool to implement the SLM programme. The contractual arrangements and a poor history of working together in some places led to a lack of trust between primary care and DHB senior leaders, which significantly affected any attempts to change funding structures in the PHO services agreement during the implementation of the SLM programme.

Further, a lack of sponsorship from the MoH ELT limited DHBs engagement in the programme. The SLM improvement plan was submitted as part of the DHB annual plan. However, alliances were not involved in DHB annual planning and development of the SLM plans was occurring in parallel. The SLM plans required signatures of alliance members as a proxy to demonstrate an integrated approach, whereas DHB annual plans did not have such a requirement. This parallel process disconnected implementation of the SLM programme from DHB planning processes.

The disconnection was exacerbated with PHOs receiving incentive funding to support the implementation of the SLM programme, albeit this funding was re-purposed from the PHO Performance Programme. The incentive funding provided the motivation for PHOs to be engaged in the SLM programme and ensured that the SLM plan and quarterly report were provided to the MoH. However, there was a lack of transparency on how this funding was used by PHOs and this created mistrust with DHBs and other alliance partners.

The dichotomy of the accountability-based health targets and the system performance approach of the SLM programme created tension in the system. DHBs, PHOs and the MoH struggled to create an alignment between the two. The allocation of resources (at DHBs and the MoH) and efforts by senior leaders continued to favour the achievement of health targets, particularly if they had a financial incentive attached.

The lack of central sponsorship also made the SLM programme fragile. The institutional knowledge about the genesis of IPIF and the SLM programme and its achievements were lost with changes in government, policies, and people involved in the implementation. The turnover of staff required constant re-education on the SLM programme, its philosophy, vision, and outcomes. The lack of central sponsorship and inadequate resourcing also led to difficulty in sustaining the programme through future policy changes. People leading policy development or those engaging with Ministers often did not understand the SLM programme and there was no impetus for them to seek this knowledge or consider the programme in policy changes.

The level of success the SLM programme had was down to the enthusiasm of individuals who believed in its philosophy and that the approach had potential to make a real difference to patient experience of care and health outcomes. They wanted to engage with integrated care and the SLM programme provided them a rare opportunity to do so. The distributed autonomy of the SLM programme (new power approach) was at odds with the command and control (old power approach) of central agencies.

### Evaluation of the SLM programme

Two studies have evaluated the SLM programme [13,14,15].

The first study examined the internal and external conditions that contributed to the successful implementation of the SLM programme [13]. The reviewers interviewed 50 managers and clinicians who were directly involved in the implementation of the SLM programme during 2018 (21 from DHBs, 27 from PHOs and two from other non-governmental organisations). They analysed SLM plans from the first three years of implementation across 18 implementation sites. Qualitative Comparative Analysis was used to analyse variation in implementation success and to identify the underlying DHB/PHO conditions that contributed to successful implementation. The authors reported that high performing districts had collaborative and inclusive planning processes that involved a broad range of stakeholders from primary and hospital care. The study concluded that successful implementation of the SLM programme required a strong platform of an alliancing way of working, positive, and high-trust relationships between organisations, and robust improvement processes that enabled continuous learning. The authors found little evidence that engaging with the SLM programme improved alliancing and high trust relationships, these were the pre-conditions required for successful implementation.

The second study (a doctoral thesis by KMS) investigated and reported on the key elements and contextual factors associated with success in the implementation of the SLM programme [14,15]. The research used the realist logic of enquiry that involved theory gleaning from the SLM programme, literature review, and evidence from those working in the health system. The latter involved interviews with senior system leaders (n = 12), two workshops with senior clinical and operational leaders, and online survey with those involved in the implementation of the SLM programme in DHBs and PHOs (n = 51) [14]. The study found that while the international evidence and intervention logic of the programme was robust, the underlying assumption that alliances would step up and improve their capacity and capability necessary to implement the programme was flawed. There were 10 key elements and five contextual factors as pre-conditions that were necessary to support the successful implementation of the SLM programme [15]. The study confirmed that the presence of trust among senior system leaders arising from a positive history of working together led to sharing of power by funders, and sustained trust between senior system leaders. Sustained trust among these leaders enabled agreement on shared vision and goals for their local system and a commitment to work towards these through an alliancing way of working. The agreement and commitment among senior system leaders resulted in a high functioning or mature alliance. The key mechanism of trust built and nurtured over time increased the maturity of alliances and increased the chances of success with implementation of the SLM programme. [14,15].

### The current state of the SLM programme

The incoming government in 2017 retired the public reporting of health targets. Under the direction of the new Minister of Health, the NZ Health Quality and Safety Commission, along with the MoH re-purposed the SLM programme as the system accountability framework under the new name of Health System Indicators (HSIs). The improvement philosophy was retained, however there were changes to the high-level outcome measures and DHB performance of the HSIs was publicly reported. This change lost momentum and trust of those involved in the development and implementation of the SLM programme as the clinical and sector leaders were not consulted on the change of approach and the measure set. Primary care leaders did not agree with the change in design from one of system performance to a DHB accountability framework. The MoH was unable to successfully negotiate these changes with PHOs in their national contract.

The 2020 reforms introduced the Pae Ora (Healthy Futures) Act 2022 and established Health New Zealand, the new entity that replaced 20 DHBs. The core objectives of the Act are to protect, promote and improve the health of New Zealanders, achieve health equity in particular for Māori, and to build towards pae ora (healthy futures) for all citizens [16].

The reforms introduced new accountability and governance arrangements, one of which was localities (geographically defined areas for the purpose of arranging services). The Act required Health New Zealand to determine localities in consultation with local authorities and iwi-Māori partnership boards and develop a locality plan for each locality. The locality plan would set out the priority outcomes and services for the locality and would be developed in consultation with consumers, communities and social sector agencies. The introduction of localities significantly disrupted the work of health alliances and many districts either disestablished their alliance or put it in abeyance. The Pae Ora reforms focused on top-down structural and legislative changes and did not consider the key enablers and pre-conditions needed to achieve the core objectives. The disruption caused by the reforms and the re-purposing of the SLM programme (as the HSIs) led to a loss of high-trust relationships between the MoH and senior primary care leaders and resulted in a loss of momentum on the implementation of HSIs. The incentive payment for PHOs remains as per the SLM programme.

The new government in 2023 re-introduced five health targets with public reporting every quarter [17]. Since 2023 Health New Zealand has been undergoing numerous internal organisational changes including the disestablishment of the Board and changes in the executive leadership teams. The current government has paused the work on localities as it considers further changes to the Pae Ora Act. Some of the districts are reviving their alliances to progress the integrated care work programme.

## Discussion

The concept of using whole-of-system measures to measure health system performance has been implemented in several health care systems internationally; for example in Sweden, Wales and the United States [18].

In 2007, the Institute of Health Improvement (IHI) published a white paper that described and promoted the use of a small set of whole-of-system measures that were not disease- or condition- specific, to measure health system performance and align improvement initiatives across the continuum of care [19]. The whole-of-system measures approach aimed to support strategic quality improvement planning and complement the traditional performance management metrics used in health systems [19]. This white paper formed the blueprint for New Zealand’s SLM programme, however the SLM programme went beyond the aspirations of the white paper. According to Chalmers, Ashton and Tenbensel [20], the New Zealand SLM programme was unique in two ways. First, it attempted to steer a middle course between the extremes of traditional health targets and pay-for-performance policy tools focussed on single organisations, and broad population health outcomes influenced by wider social sector services that are beyond the sole influence of health systems. Second, the emphasis was on a collaborative way of working using alliances rather than a financial and reputational carrots-and-sticks approach. Chalmers, Ashton and Tenbensel [20] acknowledged that no other country ‘has attempted to implement such an alliance-based approach to health system improvement at a national level’ [p 4].

The two evaluations of the SLM programme identified similar key enablers and pre-conditions needed for collaborative networks such as alliances to successfully implement a whole of system performance framework and sustain their efforts. Successful implementation of such policies relies less on meeting milestones or targets and more on cultivating and nurturing high-trust relationships, developing the capacity and capability for change, and iterative planning and practice cycles to shift behaviours of system agents.

We believe the experiment to implement the SLM programme using alliances showed the potential of a new approach to a whole of system performance improvement. While a lack of necessary pre-conditions caused variability, there was a momentum for change in response to the improvement philosophies and a willingness for sector leaders to engage through alliances. The critical gap was sponsorship and leadership from the centre. Both evaluations showed that an understanding of key elements and contextual factors by senior leaders across the system was needed to increase the chances of success with the implementation of the SLM programme. The role of the MoH was to create the right conditions for collaboration to flourish. Sponsorship and leadership from the MoH ELT were necessary to coalesce senior system leaders and empower them. Without this sponsorship, it was difficult to sustain and progress the transformative change desired by the SLM programme.

The journey of the SLM programme also highlighted the high political salience for health systems, which makes it difficult for governments and the health system to solely embrace an improvement-based system performance framework that does not complement a traditional accountability framework to meet legislative and Cabinet requirements and to assure external stakeholders, including the public.

## Conclusion

While the intervention logic for the SLM programme was in line with the international evidence and had the support of sector leaders, the programme lacked institutional and political support and adequate resourcing, making it unsustainable and vulnerable to changes in government and policies. Sponsorship and leadership from the centre are critical to coalesce system actors to collaborate on integrated care initiatives and to set up collaborative networks with a clear vision and the pre-conditions necessary to deliver on the vision. These pre-conditions include cultivating and nurturing high-trust relationships, developing the capacity and capability for change, and iterative planning and practice cycles to shift behaviours of system agents.

**Key Lessons:**

- Integrated care initiatives need sponsorship and commitment from senior leaders at the executive level to coalesce system actors and achieve the desired vision. This requires chief executives and senior clinical leaders across primary and hospital care to agree on an integrated work programme, monitor its progress, and to champion and communicate the vision and achievements within their organisations.
- Integrated care initiatives require adequate and appropriate resourcing to sustain the effort required over time. This includes dedicated teams with appropriate skills and experience, technology to share and analyse patient data, and dedicated funding across the system to implement the initiatives.
- Careful attention must be paid to system structural issues that militate against integrated care initiatives or create perverse incentives for health system actors. The DHB-PHO contractual arrangements militated against the implementation of the SLM programme. While the incentive funding acted as an enabler for PHOs, it acted as a disincentive for the DHB implementation teams.
- An improvement-based system performance framework cannot be solely used to measure system performance. An accountability framework is also needed to meet legislative and Cabinet requirements. One framework cannot be used for both purposes. For example, the SLM programme and the health targets served different purposes, with the former being used to improve whole of system performance through integrated care, and the latter fulfilled Cabinet expectations to demonstrate public accountability on hospital and PHO performance at a district level. Combining these (with the HSI approach) did not deliver on either of its objectives.
- Collaborative networks are a useful mechanism to implement a system performance framework, however these need to be set up with a clear purpose and vision from the centre, and with the pre-conditions necessary to deliver on the vision. Successful implementation of the SLM programme depended on alliance maturity, more mature alliances had a clear vision and goals, had high-trust relationships nurtured with a positive history of working together and therefore provided a strong platform for a collaborative way of working.
- Trust and behaviours in collaborative networks cannot be mandated from the centre but must be created and sustained by local senior system leaders. The role of the centre is to create the conditions that allow collaboration to flourish. The role of a senior clinician and a senior manager with a shared philosophy working together to act as relationship facilitators and continuously promote an improvement philosophy with the alliances was critical in the implementation of the SLM programme.

## Additional File

The additional file for this article can be found as follows:

10.5334/ijic.9043.s1 Figure A. Simplified NZ health system structure – Before 2021 reforms.

## References

[1] Ministry of Health. New Zealand Health System 2018. Available from: https://www.health.govt.nz/new-zealand-health-system.

[2] Hood C. A public management for all seasons? Public Administration. 1991;69(Spring):3–19. DOI: 10.1111/j.1467-9299.1991.tb00779.x

[3] Gauld R. New Zealand’s post-2008 health system reforms: Toward re-centralization of organizational arrangements. Health Policy. 2012;106(2):110–3. DOI: 10.1016/j.healthpol.2012.03.018 22502934

[4] Pye S. PHO Performance Programme: Six years on. Best practice (Dunedin, NZ). 2011;(41):40–9.

[5] Goodyear-Smith F, Ashton T. New Zealand health system: Universalism struggles with persisting inequities. The Lancet. 2019;394(10196):432–42. DOI: 10.1016/S0140-6736(19)31238-3 31379334

[6] Timmins N, Ham C. The quest for integrated health and social care – A case study in Canterbury, New Zealand. The King’s Fund; 2013.

[7] Gauld R. The theory and practice of integrative health care governance. Journal of Integrated Care. 2017;25(1):61–72. DOI: 10.1108/JICA-10-2016-0035

[8] bpac NZ. The Integrated Performance and Incentive Framework (IPIF): What has changed and how does it affect primary care? Best Practice Journal. 2014;62.

[9] Ashton T. Measuring health system performance: A new approach to accountability and quality improvement in New Zealand. Health Policy. 2015;119:999–1004. DOI: 10.1016/j.healthpol.2015.04.012 25979415

[10] Obucina M, Harris N, Fitzgerald J, Chai A, Radford K, Ross A, et al. The application of Triple Aim framework in the context of primary healthcare: A systematic literature review. Health Policy. 2018;122(8):900–7. DOI: 10.1016/j.healthpol.2018.06.006 29935730

[11] Stiefel M, Nolan K. A Guide to Measuring the Triple Aim: Population health, Experience of care, and Per capital cost. Cambridge, Massachusetts: Institute for Healthcare Improvement; 2012.

[12] PHO Services Agreement: Version 6.4. Wellington: Te Whatu Ora; 1 July 2022. Available from: https://www.tewhatuora.govt.nz/for-health-providers/primary-care-sector/primary-health-organisation-services-agreement/.

[13] Tenbensel T, Silwal PR, Walton L, Ayeleke RO. New Zealand’s Integration- Based Policy for Driving Local Health System Improvement – Which Conditions Underpin More Successful Implementation? International Journal of Integrated Care. 2021;21(2):8. DOI: 10.5334/ijic.5602 PMC8064288 33976597

[14] Sharma KM. Implementation of large-system transformation initiatives in the New Zealand health system. Wellington: Victoria University of Wellington; 2021. DOI: 10.26686/wgtn.15130413.v1

[15] Sharma KM, Jones PB, Cumming J, Middleton L. Key elements and contextual factors that influence successful implementation of large-system transformation initiatives in the New Zealand health system: a realist evaluation. BMC Health Services Research. 2024;24(1):54. DOI: 10.1186/s12913-023-10497-5 38200522 PMC10782523

[16] Pae Ora (Healthy Futures) Act 2022, Stat. 2022 No 30 (14 June, 2022).

[17] Health targets will deliver better outcomes for New Zealanders [press release]. https://www.beehive.govt.nz/release/health-targets-will-deliver-better-outcomes-new-zealanders. New Zealand Government, 8 March 2024.

[18] Doolan-Noble F, Lyndon M, Hau S, Hill A, Gray J, Gauld R. How well does your healthcare system perform? Tracking progress toward the triple aim using system level measures. NZMJ. 2015;128(1415). 26117512

[19] Martin L, Nelson E, Lloyd R, Nolan T. Whole system measures: IHI innovation series white paper. Cambridge, Massachusetts: Institute for Healthcare Improvement; 2007.

[20] Chalmers L, Ashton T, Tenbensel T. Measuring and managing health system performance: An update from New Zealand. Health Policy. 2017;121(8):831–5. DOI: 10.1016/j.healthpol.2017.05.012 28610840

