# Supplementary figures and images for: The New Zealand System Level Measures Programme – a New Policy to Implement a Whole of System Performance Framework Using Health Alliances
Source: Int J Integr Care. 2025 Nov 26;25(4):16. doi: 10.5334/ijic.9043 (PMC12662163; doi:10.5334/ijic.9043)

Figure A: Simplified New Zealand Health System Structure – Before 2021 reforms

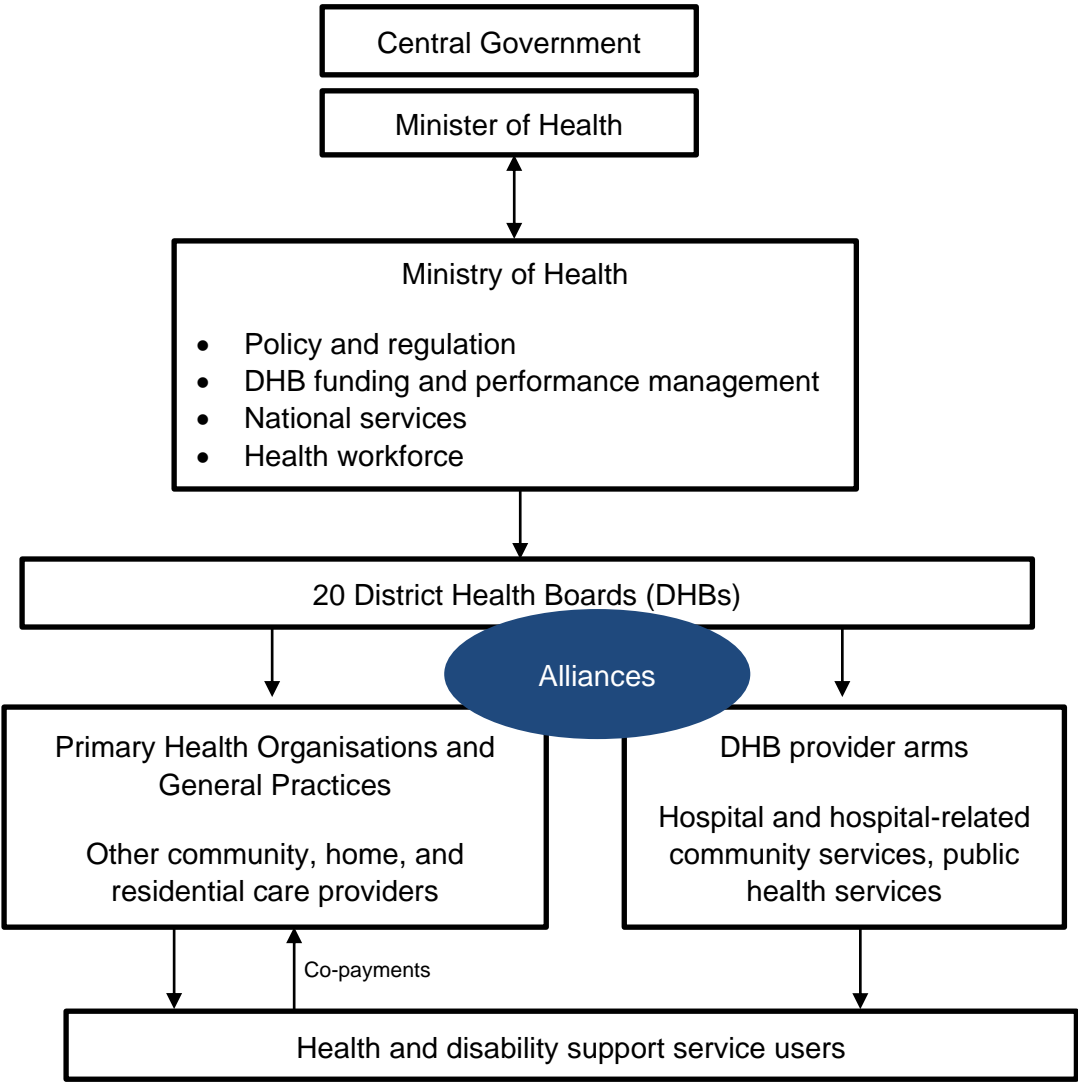

Supplement: Figure A. — Simplified NZ health system structure – Before 2021 reforms. [file ijic-25-4-9043-s1.pdf]
